# Data-driven characterization of walking after a spinal cord injury using inertial sensors

**DOI:** 10.1186/s12984-023-01178-9

**Published:** 2023-04-29

**Authors:** Charlotte Werner, Meltem Gönel, Irina Lerch, Armin Curt, László Demkó

**Affiliations:** 1 Spinal Cord Injury Center, Balgrist University Hospital, Zurich, Switzerland; 2 Rehabilitation Engineering Laboratory, ETH Zurich, Zurich, Switzerland

**Keywords:** Digital health, Inertial sensors, Rehabilitation, Gait analysis, Spinal cord injury

## Abstract

**Background:**

An incomplete spinal cord injury (SCI) refers to remaining sensorimotor function below the injury with the possibility for the patient to regain walking abilities. However, these patients often suffer from diverse gait deficits, which are not objectively assessed in the current clinical routine. Wearable inertial sensors are a promising tool to capture gait patterns objectively and started to gain ground for other neurological disorders such as stroke, multiple sclerosis, and Parkinson’s disease. In this work, we present a data-driven approach to assess walking for SCI patients based on sensor-derived outcome measures. We aimed to (i) characterize their walking pattern in more depth by identifying groups with similar walking characteristics and (ii) use sensor-derived gait parameters as predictors for future walking capacity.

**Methods:**

The dataset analyzed consisted of 66 SCI patients and 20 healthy controls performing a standardized gait test, namely the 6-min walking test (6MWT), while wearing a sparse sensor setup of one sensor attached to each ankle. A data-driven approach has been followed using statistical methods and machine learning models to identify relevant and non-redundant gait parameters.

**Results:**

Clustering resulted in 4 groups of patients that were compared to each other and to the healthy controls. The clusters did differ in terms of their average walking speed but also in terms of more qualitative gait parameters such as variability or parameters indicating compensatory movements. Further, using longitudinal data from a subset of patients that performed the 6MWT several times during their rehabilitation, a prediction model has been trained to estimate whether the patient’s walking speed will improve significantly in the future. Including sensor-derived gait parameters as inputs for the prediction model resulted in an accuracy of 80%, which is a considerable improvement of 10% compared to using only the days since injury, the present 6MWT distance, and the days until the next 6MWT as predictors.

**Conclusions:**

In summary, the work presented proves that sensor-derived gait parameters provide additional information on walking characteristics and thus are beneficial to complement clinical walking assessments of SCI patients. This work is a step towards a more deficit-oriented therapy and paves the way for better rehabilitation outcome predictions.

## Background

Depending on the severity and location of the lesion, spinal cord injury (SCI) causes heterogeneous deficits [1]. The most consistently appearing consequence is a change in the sensorimotor function [2], leading to impairments in the function of the legs, arms, or whole body. Because of recent advances in the acute management and prevention of secondary injuries, an increasing number of SCIs are being classified as incomplete [3, 4]. An incomplete injury refers to remaining sensor or motor function below the level of injury. This incomplete injury allows for a significant change in neuroplasticity, with a partial or full locomotor recovery [5]. Indeed, approximately 70% of the incomplete SCI patients will regain some ambulatory walking function [6]. However, most of the patients who regain mobility walk with deficits. An SCI gait is typically described with a reduced speed, changes in gait phase durations, and impairments in gait quality and balance [1].

Measurement tools with good clinimetric properties are essential to assess gait deficits comprehensively and to track the impact of interventions during rehabilitation on locomotion recovery [1, 7]. In the current clinical routine, walking capacity is mainly assessed using standardized gait tests. Examples for functional gait tests are the ten-meter walking test, the timed-up-and-go test, and the six-min walking test (6MWT) [8]. In the 6MWT the distance that the patient is able to walk within 6 min is measured, thus, the patients’ sustained walking speed is assessed. And indeed, walking speed has been described as the most responsive to improvement in walking capacity [9]. However, this quantitative test does not give any insights into the patient’s underlying impairments [10] and compensatory mechanisms [11]. To assess these, gait laboratories using marker-based motion capture are currently considered as the gold standard. They provide a detailed gait analysis with both spatiotemporal and kinematic parameters. However, their main drawback is that the assessments are restricted to the necessary laboratory environment, and the related expenses, time, and expertise required.

Wearable sensors such as inertial measurement units (IMUs) could become a compromise between clinical walking tests and gait laboratories. With advances in sensor technology and accessibility of these devices, they are becoming increasingly popular and have the potential to revolutionize clinical research as well as established clinical assessments [12]. The sensor units are affordable, easy to use, and do not add any burden to the patient [13]. For SCI patients, data derived from IMUs could provide additional information during a quantitative walking test by describing the gait pattern and thus capturing the gait deficits objectively. Given the relatively long duration of the 6MWT, typical spatiotemporal parameters as well as metrics related to fatiguability and quality of the gait can be gathered for analysis. To this date, most of the research using wearable inertial sensors during the 6MWT were pilot, proof-of-concept, validation and feasibility studies in mostly multiple sclerosis, stroke, Parkinson’s disease, and chronic obstructive pulmonary disease populations as summarized in the recent review of Storm et al. [14].

One of the challenges of using technology-aided assessments is the plethora of generated outcome measures which often have a high covariance [15] and are usually difficult to interpret for clinicians [16]. To avoid redundancy and facilitate interpretation, approaches such as principal component or factor analysis can be used to identify and group relevant outcome metrics into domains, e.g. rhythm and symmetry of gait. This approach has been applied to elderly, and Parkinson’s disease populations, as well as in idiopathic fallers using gait metrics generated from an electronic walkway [17]. A data-driven selection of relevant sensor-derived gait parameters for a comprehensive characterization of walking after a spinal cord injury using an extensive dataset is still missing. So far, studies using IMUs to characterize walking in individuals with SCI focused mainly on the validity of the sensor-derived metrics [18, 19], the test-retest reliability [20], or sensor-derived metrics were manually selected to compare different walking conditions [21–23].

Current clinical assessments have mainly two purposes: to track the patient’s current status objectively but also to serve as a foundation for rehabilitation outcome predictions by clinicians. Technology-aided assessments could further enhance such rehabilitation outcome estimations. As an example, Kanzler et al. [15] have shown that including digital health metrics for the prediction of upper limb rehabilitation outcomes in multiple sclerosis remarkably increased the accuracy of the model by 10%. Whether sensor-derived gait parameters could similarly help to predict if a patient will improve his or her walking capacity has not yet been investigated for SCI patients. Especially in this heterogenous patient cohort, better prediction models of recovery profiles are needed to manage the patient’s expectations better and to improve personalized and targeted treatment plans further.

The project aimed to identify sensor-derived gait parameters that complement a standardized walking test. A dataset of patients with an incomplete SCI and healthy controls performing a 6MWT while wearing a sparse sensor setup of one IMU attached to each ankle has been acquired. Further, demographics and clinical scores were collected to bring the sensor-derived gait parameters into context with established clinical characteristics. A subset of the participants with SCI was performing the instrumented 6MWT several times during their course of rehabilitation. This longitudinal dataset allowed the training of a prediction model to estimate whether a patient will improve the walking capacity or not. The hypotheses of this project were that (i) sensor-derived gait parameters can identify gait deficits not captured by the walking speed, such as compensatory movements, and (ii) including sensor-derived parameters as predictors will improve the classification accuracy of whether a patient will improve the walking capacity in the future. A data-driven approach using signal processing and machine learning techniques to extract and select the relevant sensor parameters was used to address these two research hypothesis.

## Methods

### Subjects

The participants of this study were individuals with an incomplete SCI, undergoing either a stationary or ambulatory rehabilitation program. Patients with all neurological levels of injury were included if they were older than 18 years and were able to walk for at least 10 m without physical assistance of a therapist. However, the use of walking aids such as braces, walkers, crutches, canes or similar was allowed. Participants had to be excluded if comorbidities affecting their gait, such as orthopedic problems, were present. In addition, data from neurologically unimpaired participants was collected as reference data of healthy controls. Similarly, these participants had to be older than 18 years and without any orthopedic problems. The measurements were approved by the ethics committee of the Canton Zurich (BASEC No. 2022-00730) and merged with a previously recorded dataset, including data from healthy controls (KEK- ZH No. 2013-0202). All measurements were performed at the University Hospital Balgrist in accordance with the standards of the Declaration of Helsinki and Good Clinical Practice guidelines.

### Protocol and data collection

Clinical scores were collected (if available) from the electronic medical record system for the participants with SCI. From the American Spinal Injury Association impairment scale (AIS), the lower extremity motor score (LEMS), the neurological level of injury (NLI) and the completeness of the injury were retrieved. Further, the Spinal Cord Independence Measure (SCIM) [24], the Mobility domain of the SCIM, the Walking Index for Spinal Cord Injury (WISCI) [25], and the days since injury were compiled. A patient was assumed to be in a chronic stage if the injury happened more than 365 days ago. Demographic information has also been gathered for all participants, such as age, weight, height, and sex.

All participants performed a 6MWT at their self-selected walking speed. The subjects were asked to walk safely but as quickly as possible along a hallway. Rest was allowed, and patients could also use walking aids if needed. The type of walking aid used and whether the participant needed an ankle orthosis were recorded. An experienced physiotherapist administered the test as part of the rehabilitation program. A subset of the participants with SCI performed the 6MWT several times during their rehabilitation, leaving at least two weeks between two consecutive assessment sessions to track their improvement.

**Fig. 1:**
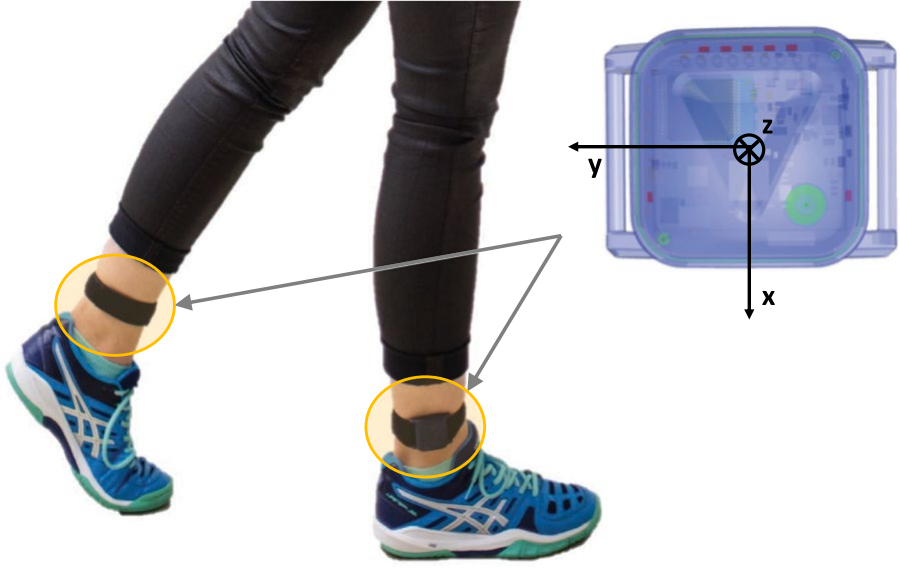
Participant wearing one inertial measurement unit attached with a flexible strap laterally above each ankle. Enlarged schematics depicts the sensor module with its local coordinate system

During the walking test, the participants had one inertial sensor unit (ZurichMOVE, Switzerland) attached with flexible straps lateral above each ankle as shown in Fig. 1. The IMU modules (MPU-9250, 35 x 35 x 12 mm, 18 g), which included a tri-axial accelerometer (range: ±16 g), a tri-axial gyroscope (range: $\pm 2000^\circ /\hbox {s}$), and a tri-axial magnetometer, recorded at a sampling frequency of 200Hz. Magnetometer data was not included in the analysis because the magnetic field is often distorted indoors. The two inertial sensor units were time synchronized via Bluetooth Low Energy.

### Data postprocessing

IMUs require appropriate post-processing to extract metrics of interest from the raw sensor data. Here, an algorithm was used that was previously developed in our group and validated specifically for the population of SCI [19]. The processing steps of this algorithm are explained in brief in the following and the collection of the extracted gait parameters are summarized in Table 1. An extensive description of the algorithm and the accuracy of the sensor-derived spatio-temporal parameters in comparison to a gold standard system can be found in our previous work [19].

**Table 1:** Description of gait parameters

Gait parameter (Statistical features)	Description
Stride duration (mean, cov, asym, d2r)	Time between two consecutive heel strikes of the same side
Step duration (mean, cov, asym, d2r)	Time between the heel strike of one side until the following heel strike of the opposite side
Swing phase (mean, cov, asym, d2r)	Relative time of the time between toe off and heel strike of the same side w.r.t. the stride duration
Double support phase (mean, cov, asym, d2r)	Relative time of the time when both feet are on the ground w.r.t. the stride duration
Stride length (mean, cov, d2r)	Distance in-between two heel strikes of the same side
Stride width (mean, cov, asym, d2r)	Maximum lateral displacement during a stride
Stride height (mean, cov, asym, d2r)	Maximum vertical displacement during a stride
Cyclogram top view (ACC, SSD, area)	Top view of the ankle endpoint trajectory
Cyclogram side view (ACC, SSD, area)	Side view of the ankle endpoint trajectory
Smoothness	Modified spectral arc length of the angular velocity of the sagittal plane
Change in speed, stride length, cadence	Slope divided by the intercept of the linear fit of the parameters over the 6 min
Speed inconsistency	Absolute value of the change in speed

**Fig. 2:**
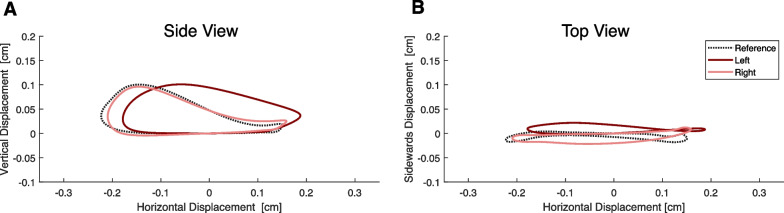
Side view (**A**) and top view (**B**) cyclograms of the ankle endpoint trajectory. Curves are shown for both the left and right sides of an exemplary SCI patient (with a clear more and less physiological side), together with the averaged reference data of healthy controls

The algorithm uses adaptive thresholds to detect individual steps and gait events based on the frequency spectrum of the data, which makes this algorithm robust across a wide range of walking speeds. More specifically, a fast Fourier transform is applied to the gyroscope data perpendicular to the sagittal plane ($\omega _z$). The first main frequency component of this frequency spectrum corresponds to the average walking cadence. Individual strides and gait events, such as the initial and final foot contacts, are identified by local peaks in the gyroscope and accelerometer data. The window width, in which these peaks are searched for, and the threshold for the minimum peak height are adapted based on the average walking cadence and the distribution of $\omega _z$, respectively. The cadence and typical gait phases, such as the swing, stance, and double support phases can subsequently be derived from these gait events.

Further, the 3D sensor trajectory for each stride is reconstructed using a typical double integration approach. The underlying concept is to integrate the acceleration data twice to obtain displacement trajectories. However, accelerometers measure not only movement acceleration, but also gravity, which needs to be subtracted prior to integration. Hence, the orientation of the sensor is estimated using the magnetometer-free approach of Seel et al. [26]. The drift in the inclination angle can be corrected by fusing the accelerometer and gyroscope data. Using this sensor orientation, the sensor data can be transformed from the local, moving coordinate system into a global, fixed coordinate system. In this fixed coordinate system, the gravitational component can be subtracted from the vertical axis of the accelerometer data. After receiving the pure movement acceleration, this data is integrated twice for each stride using a trapezoidal integration method to obtain the 3D trajectories. Since a magnetometer-free approach was chosen, the orientation estimation suffers from a drift around the heading angle, which is addressed by rotating the data towards the main movement direction as described by Trojaniello et al. [27]. In addition, the sensor data suffers from thermo-mechanical noise, which results in a second-order drift when being integrated twice. Smartly chosen boundary conditions address this issue, such as the “zero-velocity-update” during mid-stance modified for the ankle sensor placement. Spatial parameters like the stride length, height (maximum vertical displacement), and width (maximum sidewards displacement) can then be extracted from the sensor trajectory. Further, the walking speed is derived from the stride duration and length.

**Fig. 3:**
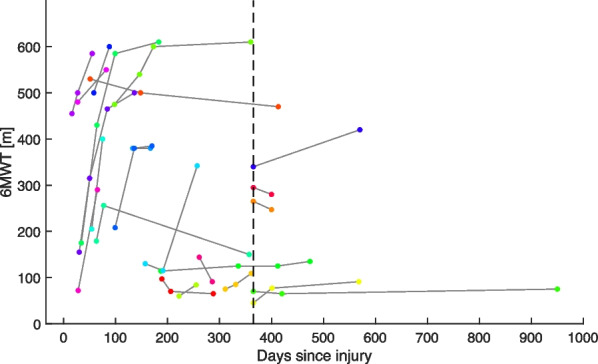
6MWT outcome of the subset of 23 patients with SCI that performed the assessment at least twice during rehabilitation. Data points corresponding to the same patient are connected and displayed with respect to their time since injury. The dotted line indicates the beginning of the chronic phase

**Fig. 4:**
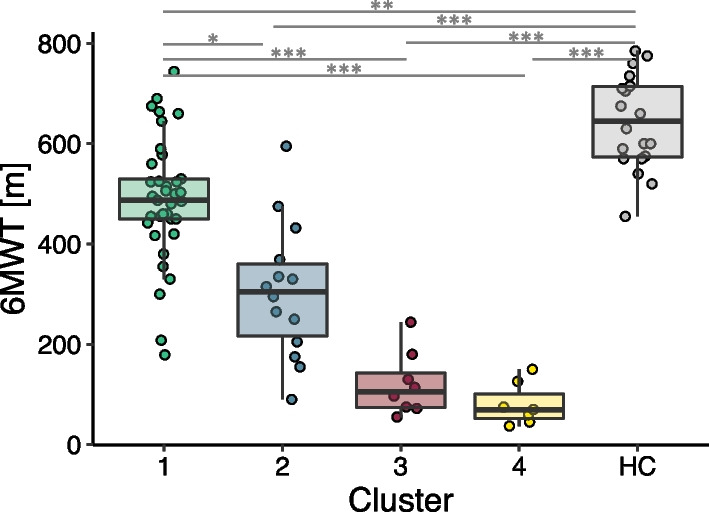
6MWT performance of the 4 clusters and healthy controls (HC). Significant differences are indicated by *(< 0.05), ** (< 0.01) or ***(< 0.001)

All gait parameters *X* (stride duration, step duration, swing phase, double support phase, stride length, stride width, stride height) were extracted for both legs and all strides. Statistical features were computed for these, such as the mean, coefficient of variation, the asymmetry and difference to reference. The coefficient of variation (cov) is defined as the standard deviation $\sigma$ divided by the mean $\overline{X}$ of all strides during the 6MWT.

1 $$\begin{aligned} cov = \frac{\sigma }{\overline{X}}*100\% \end{aligned}$$

Further, the asymmetry (asym) between both sides was computed with the symmetry index [28]:

2 $$\begin{aligned} asym = \frac{|\overline{X}_{left}-\overline{X}_{right}|}{0.5*(\overline{X}_{left}+\overline{X}_{right}))}*100\% \end{aligned}$$

And the difference to reference (d2r) was computed as the difference to the gait parameter of healthy controls $\overline{X}_{ref}$ interpolated to the same walking speed.

3 $$\begin{aligned} d2r = \frac{\overline{X}-\overline{X}_{ref}}{\overline{X}_{ref}}*100\% \end{aligned}$$

In addition to the spatiotemporal gait parameters, ankle cyclograms have been derived from the 3D sensor trajectory by subtracting the endpoint’s displacement with reference to each stride’s starting point. This processing step results in 3D enclosed shapes, as shown for one example patient in Fig. 2. After scaling and centering these top view and side view cyclograms, the shape can be compared to a physiological reference shape with the sum of squared differences (SSD) as described by Awai et al. [29]. The advantage of this method is that it is independent of the stride length. An SSD of 0 would indicate no difference between the cyclogram of the participant and the physiological reference. Furthermore, the within-subject cycle-to-cycle consistency of these cyclograms was quantified by the angular component of coefficient of correspondence (ACC) as described by Field-Fote et al. [30]. The range of ACC goes from 0% (no consistency) to 100% (perfect consistency). In addition, the area enclosed in these cycles was derived. The SSD, ACC, and the area were computed for the side and top view cyclograms.

**Fig. 5:**
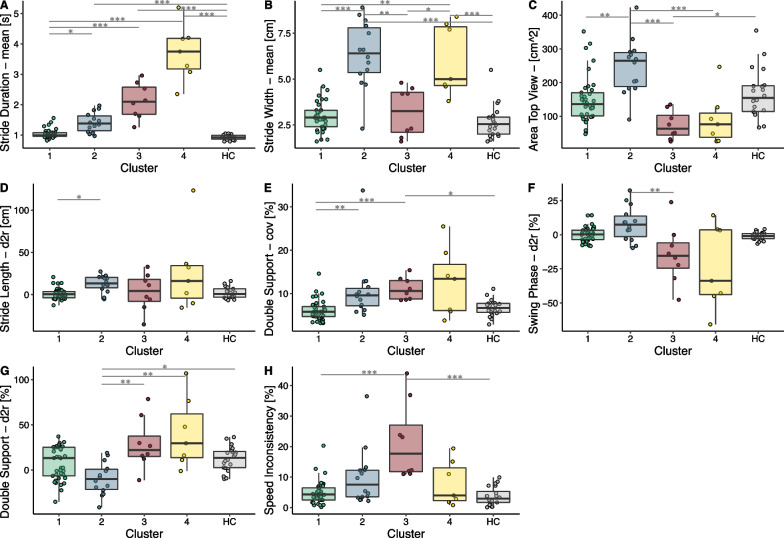
Selected gait parameters (**A**–**H**) shown for the 4 clusters and healthy controls (HC). Significant differences are indicated by *(< 0.05), ** (< 0.01) or ***(< 0.001)

**Fig. 6:**
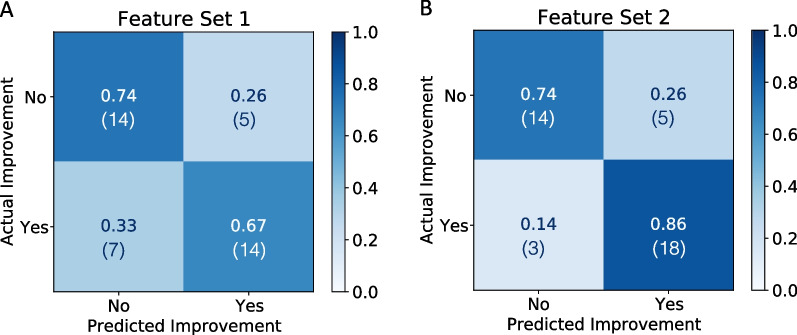
Classification performance of “Feature Set 1” (**A**) and “Feature Set 2” (**B**) of the prediction whether a participant will improve the walking capacity. Confusion matrices show both the normalized (and absolute) counts for each class

The movement smoothness was quantified by the frequency spectrum of the sagittal angular velocity. More specifically, the modified spectral arc length was calculated according to Balasubramanian et al. [31], because this method is less prone to differences in the duration of the movement and can be applied for cyclic movements like gait [32].

Further, the change in speed, stride length, and cadence over the 6 min was computed as a measure of fatigue. The change was defined as the slope of a linear fit of the speed, stride length, and cadence divided by the intercept of this linear fit. In addition, the speed inconsistency was computed as the absolute value of the change in speed.

**Table 2:** Demographics and clinical characteristics of the participants

Cohort	SCI	Healthy
Number	66	20
Age	55.6 ± 15.1 years	58.6 ± 11.4 years
Sex	28.8% female	20% female
BMI	24.9 ± 4.9 kg/m$^2$	24.0 ± 3.86 kg/m$^2$
6MWT	362 ± 195 m	644 ± 93 m
Chronicity	34.8% acute	
Diagnosis	48.5% traumatic	
AIS	B: 3	
	C: 2	
	D: 50	
	NA: 11	
NLI	Cervical: 25	
	Thoracic: 22	
	Lumbar: 12	
	Sacral: 2	
	NA: 5	
LEMS	41.9 ± 9.5	
SCIM	76.2 ± 21.1	
SCIM Mobility	30.2 ± 10.6	
Walking Aid	47.0% with	
Orthosis	15.2% with	
WISCI II	13.1 ± 5.4	

Values are presented as mean ± standard deviation. *BMI* body mass index, *AIS* ASIA Impairment scale, *NA* not assessed, *NLI* neurological level of injury, *LEMS* lower extremity motor score, *SCIM* spinal cord independence measure, *WISCI* walking index for spinal cord injury

To reduce redundant information, gait parameters were only used for the analysis from the more impaired side (if available for both sides), which was defined as the side with the lower LEMS. The right side was taken for the healthy controls, and if both sides had the same LEMS score.

### Statistical analysis

#### Identifying gait clusters

A cluster analysis was performed on the gait parameters of the first 6MWT of all participants with SCI to identify patients with similar gait characteristics. First, a principal component analysis was executed on the scaled and centered gait parameters to reduce the high-dimensional dataset. The number of principal components (PCs) for the clustering was selected based on the cumulative explained variance. To identify the optimal number of clusters a hierarchical clustering using Ward’s criterion was performed. A k-means clustering on the PCs assembled the SCI participants into distinct groups. A variance analysis of demographic data and clinical scores identified significant differences in the cluster composition. In particular, a Kruskal-Wallis test was chosen for the continuous variables (e.g., SCIM) and a Fisher test for the categorical variables (e.g., percentage of acute patients) due to the non-normality of the data. Further, the most discriminating gait parameters between the clusters were identified to characterize the walking pattern of the different clusters. More specifically, the first five gait parameters that contributed the most to each PC were selected. This set of parameters was further reduced to a core set of gait metrics, by only keeping parameters that showed a significant difference between the clusters (Kruskal-Wallis, $\alpha = 0.05$) and by eliminating parameters that highly correlated with another parameter (Pearson correlation coefficient $> 0.9$) to reduce redundant information. A post-hoc test (Dunn test, $\alpha = 0.05$) identified significant differences between the clusters in this core set of gait parameters and allowed the comparison of the clusters to healthy controls.

#### Prediction of improvement in walking capacity

To predict whether a patient will improve the walking capacity significantly in the future, a machine learning model has been trained on the data of participants that performed the 6MWT at least twice. The dependent variable of the model was whether the participant improved in the 6MWT from the “present” to “future” assessment more than the standard error of measurement (SEM), which was reported to be 16.5 m for SCI [33]. Any increase above this SEM was assumed to be an actual change in walking capacity rather than measurement noise. In other words, a binary random forest classifier was trained to predict whether a patient will improve above the SEM or not until the following 6MWT assessment.

**Fig. 7:**
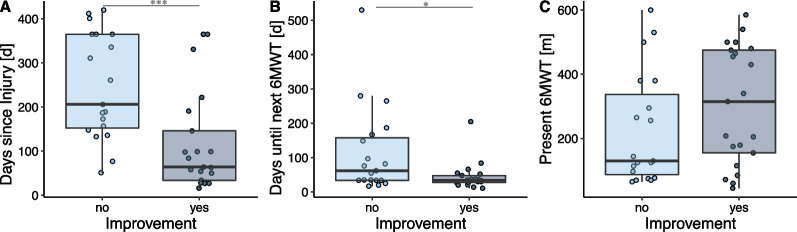
“Feature Set 1”: Days since injury (**A**), days until next 6MWT (**B**) and present 6MWT (**C**) grouped by whether the patient will improve until the next 6MWT or not. Significant differences are indicated by *(< 0.05), ** (< 0.01) or ***(< 0.001)

**Fig. 8:**
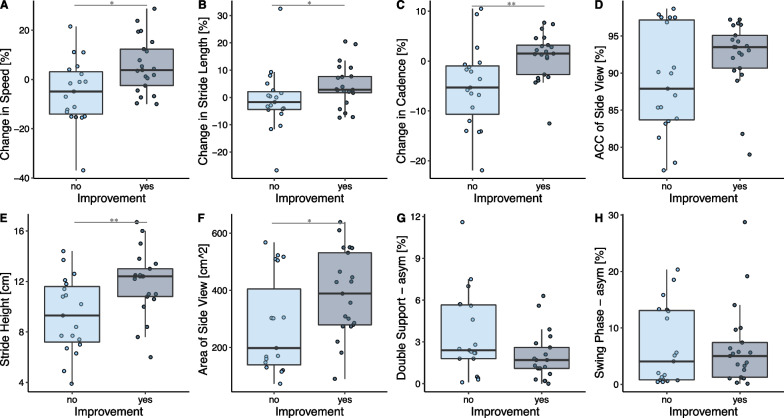
Gait parameters (**A**–**H**) of “Feature Set 2” grouped by whether the patient will improve until the next 6MWT or not. Significant differences are indicated by *(< 0.05), ** (< 0.01) or ***(< 0.001)

**Fig. 9:**
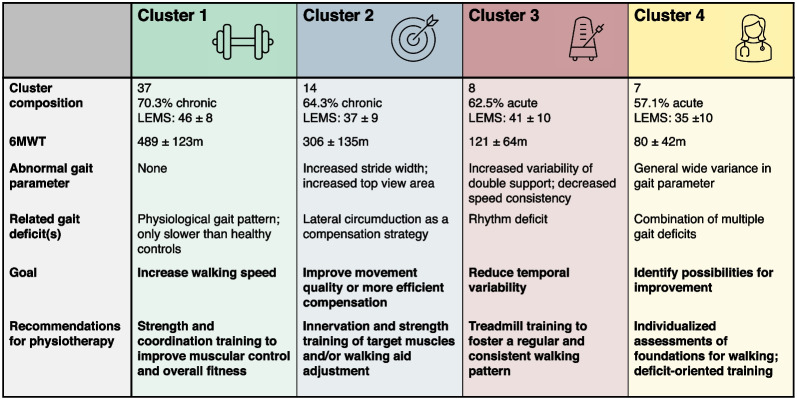
Summary of the main findings on the cluster composition, gait characteristics and recommendations. *LEMS* lower extremity motor score

Two different feature sets were used to identify whether the sensor-derived gait parameters can improve this prediction when using them as additional predictors. The first feature set only included the “present” 6MWT distance, at what time point after injury this “present” 6MWT was performed, and the number of days until the “future” 6MWT was performed. If the time point of the first 6MWT was more than 365 days after injury, all trials of this patient were shifted such that the first 6MWT was set to 365 days, because it is assumed that after this time point, the patient is in a chronic state. The second feature set additionally included all sensor-derived gait parameters from the “present” 6MWT as predictors. All features were scaled to have unit variance and centered around their respective mean. Further, redundant features (Pearson correlation coefficient > 0.9) were removed and only the first 10 most contributing features were included to avoid overfitting of the model to the training data.

The classifier was trained and evaluated in a leave-one-subject-out cross-validation procedure, which means that the classifier was trained on all trials except for the trials of one participant, and then tested on the trials of this excluded participant. This procedure was repeated until the classifier was trained and tested on all data to evaluate the model’s generalizability to unseen data. Accuracy was chosen as the evaluation metric to compare the predictive power of the two different feature sets. Accuracy was defined as the sum of true positives and true negatives divided by the total number of observations. Difference in features between improvers and non-improvers were analysed with the Kruskal-Wallis test ($\alpha$ = 0.05).

## Results

### Participants

The demographics and clinical characteristics of the 66 participants with SCI and 20 healthy controls are summarized in Table 2. Both cohorts were similar in age, sex distribution, and BMI. However, the healthy controls achieved overall longer distances in the 6MWT compared to those achieved by the participants with SCI (644±93 m vs. 362±195 m). The cohort of participants with SCI was quite heterogeneous. The first measurement was conducted in the chronic phase (> 365 days after injury) for around 65% of the SCI participants. Around half of the participants had a traumatic injury. The majority had an injury that was both sensory and motor incomplete (AIS D score), whereas the NLI ranged from cervical to sacral. On average, the participants with SCI had a LEMS of $41.9 \pm 9.5$ out of a maximum achievable score of 50 and a SCIM of $76.2 \pm 21.1$ out of 100. Focusing only on the mobility domain of the SCIM, the participants had a score of $30.2\pm 10.6$ out of 40. In terms of walking aids, 47% used some type of walking aid and 15.2% used an orthosis, which resulted in an overall WISCI II score of $13.1\pm 5.4$ out of 20.

A subset of 23 out of the 66 participants with SCI performed the 6MWT more than once at different time points during their rehabilitation: 10 were measured twice, 9 were measured three times, and 4 were measured four times. The days since injury and the 6MWT outcome for this subset of participants are shown in Fig. 3. The median time between the two measurements was 35.5 days (Inter-quartile range: 30–79).

### Characterization of the gait clusters

To identify groups of patients with similar gait characteristics, the patients were clustered on the PCs derived from the sensor gait parameters. The first four PCs of the gait parameters explained 69.4% of the variance in the data and were selected for the clustering. Four distinct clusters were obtained. Their composition in terms of demographics and clinical scores is presented in Table 3. The clusters neither differed in demographics (age, sex, BMI) nor in the diagnosis (traumatic/non-traumatic) or the chronicity of the injury. However, the clusters differed significantly in their performance in the 6MWT, which determined the cluster number ordering and is presented in Fig. 4. It was found that both the first cluster and the healthy cohort differed significantly from every other. Further, the clusters varied in the clinical scores, such as the LEMS, SCIM, SCIM Mobility, use of walking aid or orthosis, and WISCI. With a few exceptions, the motor capacity and independence measures were decreasing, and the use of walking aids or orthosis was increasing with increasing cluster number.

**Table 3:** Cluster composition

	Cluster 1	Cluster 2	Cluster 3	Cluster 4	p-value
Number	37	14	8	7	
Age	54.8 ± 15.3	60.2 ± 15.4	52.2 ± 14.3	55 ± 15.7	0.619
Sex	24.3% female	21.4% female	62.5% female	28.6% female	0.193
BMI	25.3 ± 4.6 kg/m$^2$	24.9 ± 5.1 kg/m$^2$	22.1 ± 8.2 kg/m$^2$	24.1 ± 5.1 kg/m$^2$	0.752
6MWT	489 ± 123 m	306 ± 135m	121 ± 64m	80 ± 42m	< 0.001
Chronicity	29.7% acute	35.7% acute	62.5% acute	57.1% acute	0.232
Diagnosis	48.6% traumatic	42.9% traumatic	37.5% traumatic	71.4% traumatic	0.601
LEMS	45.8 ± 7.6	37.3 ± 9.3	41.1 ± 10.3	34.7 ± 10.1	0.005
SCIM	85.2 ± 18.5	69.0 ± 16.9	60.6 ± 21.6	59.6 ± 17.6	< 0.001
SCIM Mob.	36.3 ± 7.0	27.4 ± 9.6	19.8 ± 6.6	15.6 ± 4.9	< 0.001
Walking Aid	24.3% with	57.1% with	100% with	85.7% with	< 0.001
Orthosis	10.8% with	0% with	25.0% with	57.1% with	0.006
WISCI II	16.8 ± 4.2	12.6 ± 5.4	11.0 ± 4.0	7.0 ± 1.1	< 0.001

Demographics and clinical characterization of the clusters with the corresponding p-values of the Kruskal-Wallis test. Values are presented as mean ± standard deviation. *BMI* body mass index, *AIS* ASIA impairment scale, *NLI* neurological level of injury, *LEMS* lower extremity motor score, *SCIM* spinal cord independence measure, *WISCI* walking index for spinal cord injury

Further, the clusters were compared in terms of the most relevant gait parameters, which were obtained with a feature selection procedure. More specifically, the five most important features of each PC were picked and then checked for redundancy. This procedure resulted in eight gait parameters shown in Fig. 5, where the results are displayed for each cluster and the healthy controls. Comparing the clusters to the healthy controls with respect to these eight features, it was found that cluster 1 and healthy controls did only differ in terms of their performance in the 6MWT. Cluster 2 showed a significantly higher stride width, stride duration, and an abbreviated double support phase (negative d2r). Cluster 3 had a higher stride duration, a lower top view area, and a higher variability in the double support phase. Furthermore, the speed was less consistent than in healthy controls. Cluster 4 had a longer stride duration and higher stride width than healthy controls.

When comparing the individual clusters to each other, it was found that the stride duration of cluster 1 differed significantly from those of the other 3 clusters, but no substantial discrepancy has been found between these 3 clusters. The stride widths of clusters 2 and 4 were significantly higher compared to the other two clusters. Further, the top view area of cluster 2 was significantly higher than in the other three clusters. The stride length was more extended (positive d2r) in cluster 2 than in cluster 1. The variability (cov) of the double support phase was lower in cluster 1 than in cluster 2 and 3 and the d2r of the swing phase was different in cluster 2 compared to cluster 3. More specifically, the swing phase of cluster 2 was found to be prolonged, whereas that of cluster 3 shorter than in healthy controls walking at the same speed. Accordingly, the d2r of the double support phase differed significantly between clusters 2 and 3, as well as between clusters 2 and 4. The speed was less consistent in cluster 3 than in cluster 1.

### Prediction of improvement in walking capacity

A subset (23 patients) was measured either 2, 3, or 4 times during the course of their rehabilitation. When counting two consecutive 6MWTs as one observation for the prediction model, the dataset consisted of 40 observations. Twenty-one observations were “improvement” and 19 “no improvement”, depending on whether the improvement in the 6MWT distance was greater than the SEM of 16.5 m or not, respectively.

The prediction model of whether a patient will perform better at the next 6MWT with an improvement more than the SEM was tested on two different feature sets and is presented in Fig. 6. The binary classification yielded an accuracy of 70% when the model was trained on the “present” 6MWT distance, the days since injury, and the days until the next 6MWT assessment only. Including sensor-derived gait parameters (feature set 2) improved the performance of the classifier by 10% and thus achieving an overall accuracy of 80%. The 10 most important features selected by the model were (in the order of importance): the days since injury, the change in cadence, the side view ACC, the days until the next 6MWT, the asymmetry in the double support phase, the change in stride length, the asymmetry of the swing phase, the stride height, the change in speed, and the area of the side view. How the features of set 1 and the additional sensor-derived features differed between the improvers and non-improvers is shown in Figs. 7 and 8, respectively. Most improvers were in the acute phase. And on average, there were fewer days until the next 6MWT. Interestingly, the “present” 6MWT distance did not differ significantly between the improvers and non-improvers.

From the sensor-derived features, it was found that improvers tend to have a positive change in speed, stride length, and cadence. Further, the improvers showed a higher stride height and side view area. The improvers also showed on average a slightly better cyclogram consistency (ACC side view) and a slightly lower temporal asymmetry (asymmetry of the double support and swing phase), but these last findings were not found to be significant.

## Discussion

In this work, we presented a data-driven characterization of the gait properties of patients with a SCI. The data of 66 participants with SCI and 20 healthy controls performing a 6MWT while wearing IMUs attached to their ankles was used. A subset of 23 SCI participants performed the 6MWT at least twice, with a minimum of two weeks between each assessment. Machine learning and statistical methods were used to select wearable sensor-derived outcome measures relevant (i) to identify and characterize groups of SCI patients with similar gait characteristics and (ii) to predict whether a patient will improve their walking capacity significantly in the future.

The patients with SCI included in this study represented this heterogeneous patient cohort. More specifically, patients with different levels (from cervical to sacral) and completeness (sensory and motor) of the spinal injury were measured, from both acute and chronic stages. The average performance in the 6MWT of the participants with SCI included in this study was slightly higher (362±195 m) than values of chronic SCI patients (317±22 m) found by Barbeau et al. [34]. The fact that around 35% of the participants with SCI in this study were in the acute phase and thus usually having a lower walking capacity explains the larger standard deviation.

### Characterization of the gait clusters

A clustering procedure based on sensor-derived gait parameters separated the patients into four clusters. The cluster’s composition and gait characteristics were analyzed by identifying the most relevant and non-redundant gait parameters.

Cluster 1 was the most prominent cluster, with 37 patients. The mainly chronic SCI patients in this cluster had a significantly lower performance in the 6MWT than the healthy controls but did not differ in any of the sensor-derived gait parameters. Hence, participants of this cluster walked slower but with a physiological walking pattern. Our recommendation for physiotherapy of patients in this cluster would be to improve their walking capacity by improving their intra- and inter-muscular regulation with strength training and coordination training. This should improve muscular control and overall fitness to foster speed improvement.

Participants of cluster 2 walked significantly slower than the participants of cluster 1. The most prominent walking characteristic of this cluster was the increased stride width and increased top view area, which are both indicators for a lateral circumduction and thus compensatory movements [35]. Further, the double support phase is abbreviated (d2r) in cluster 2 in comparison to reference data presumably due to the compensatory movements mainly during the swing phase leading to redistribution in the relative gait phases. This indication is further confirmed when comparing the cluster composition of cluster 2 and 3: an overall lower LEMS and a higher percentage of chronic patients was found in cluster 2. Hence, we can assume that patients of cluster 2 learned compensatory strategies that enable this group to walk faster in comparison to acute patients with better muscle scores. This is in line with the literature, as several studies show that functional improvement can occur independently from neurologic recovery by using compensatory mechanisms [6, 36]. Our recommendation for patients in this cluster is to improve their movement quality by innervation training and strength training of the target muscles rather than focusing on improving speed.

The most prominent characteristic of cluster 3 was found to be the high variability of the double support phase and the speed inconsistency. This high variability in rhythm has been shown to be a risk factor for falling [37]. We would recommend focusing on reducing mainly the temporal variability in these patients, e.g., by using robotic devices, such as the Lokomat®, or simple treadmill training. The predefined walking consistency and the many repetitions would foster a regular and periodic gait pattern towards safer walking.

Similar to cluster 2, an increased stride width was obtained in cluster 4, which again indicates a lateral circumduction and thus compensatory strategy [35]. In addition, cluster 4 was the smallest cluster with only four patients and showed a high variance, especially in the gait parameters related to the gait phases. We assume these patients suffer from multiple gait deficits, resulting in a wide variance in the gait parameters. Especially for the 57% acute SCI patients included in this cluster, we recommend an individualized assessment of the foundations for walking, such as postural control and standing stability. Then, deficits with the possibility of improvement should be identified and addressed in a personalized deficit-oriented training manner.

The main findings of this clustering including a physiotherapy recommendation for each cluster are summarized in Fig. 9.

### Prediction of improvement in walking capacity

Using the longitudinal data of a subset of patients with SCI that performed the 6MWT at least twice, predictors of whether a patient will improve in the future more than the SEM were identified. When the present 6MWT distance, the time since injury, and the days until the next 6MWT were provided as inputs to the model a prediction accuracy of 70% was achieved. It was found that improvers were mainly acute SCI patients that were measured again after a median of 34 days. Interestingly, the performance in the 6MWT of the improvers ranged from 45 m to 585 m and was not statistically different from the non-improvers, which means that both slow and fast walkers were able to improve their walking speed. This is in line with the literature where it was previously observed that the recovery of walking speed in SCI patients did not depend on the initial speed [6]. Wirz et al. demonstrated that in SCI patients, it rather depended on an inherent capacity of functional improvement irrespective of initial impairment.

Adding sensor-derived gait parameters as predictors for the binary classification model could improve the prediction accuracy by 10%, to 80%. The sensor-derived features exhibited that improvers tend to have a higher change in speed, stride length, and cadence as predictors. Since the majority of improvers were in the acute phase, we assume that these patients improve their speed, stride length, and cadence consistency, both due to becoming more familiar with the test and also because they get better in estimating their abilities over the 6 min. Further, the non-improvers showed a lower stride height and lower side view area in the present 6MWT, which might indicate weak hip and knee flexors to lift the foot. Previous studies stated that hip flexors, hip extensors, and hip abductors are determinant for ambulatory function [38, 39]. In addition, improvers tend to have a lower asymmetry in the gait phases (double support and swing phase) and higher cyclogram consistency (higher ACC), even though these observations were not significant. In summary, patients that improved their performance in the 6MWT already had a more physiological walking pattern than the non-improvers, and thus it can be assumed that the improvers mainly improved their speed and thus performance in the 6MWT by improving their overall fitness and speed consistency. Accordingly, the gait deficits of the non-improvers presumably hinder this group from improving their speed considerably.

### Limitations

The main limitation of this work is that many of the gait parameters correlate with speed, such as for example the cadence, swing phase, and stride length, which is widely known [40]. Hence, it is difficult to disentangle walking quality and speed for participants walking at different speeds. Further, the slow walking patients are often the more severely affected part of the population. We tried to address this issue by providing parameters that are less related to walking speed, e.g., the measures related to variability and symmetry. Future work focusing on walking quality should consider collecting data of patients walking at similar speeds to get rid of this effect.

Since most of the data was collected as part of the clinical routine, the assessments were unequally spaced in time. 6MWT assessments were performed approximately every four weeks on average, but especially for chronic patients, the assessments were performed less often and only when the patient was in ambulatory rehabilitation. This unequal time spacing introduced additional noise in the data that we tried to address by providing the time until the following assessment as a predictor to the model. Moreover, sensor-derived gait parameters suffer generally from estimation errors. Such errors would distort both the clustering of participants as well as the prediction model. As shown in previous work [19], the algorithm used to derive the spatio-temporal parameters showed excellent results even for individuals with SCI walking with distinct gait deficits at slow walking speeds of 0.5 m/s. Hence, the effect of estimation errors on the clustering and prediction model should be minimal.

### Clinical implications and future work

The results underline the benefit of using wearable inertial sensors during the 6MWT. Based on the sensor-derived gait parameters, different groups of patients were identified that differed not only in terms of walking speed but also in terms of quality-related gait characteristics. Future work of our group will focus on translating these findings into the clinical routine by providing an easy-to-use tool for sensor-based gait assessments, including a tablet-based gait report. Part of this report will focus on the gait parameters identified in this work. Such a tool will foster a more deficit-oriented gait therapy by using the objective gait measures and the corresponding recommendations for physiotherapy provided in this work. Furthermore, the tool will also allow to objectively track improvements in gait metrics other than pure walking speed. This would give a more comprehensive picture of the rehabilitation progress of individuals and might be beneficial to justify the continuation of therapy towards health insurance companies.

Including sensor-derived metrics in the prediction model resulted in an accuracy of 80% of the estimation whether a patient will increase his or her walking capacity. Combining such a data-driven model with the expertise and experience of clinicians would result in better expectation management of patients and more accurate definition of rehabilitation goals. Nevertheless, more work on clinical applicability of such prediction models is needed.

Moreover, the possibilities of applying this sensor-based gait analysis system go beyond the standardized 6MWT. Given the sparse sensor setup used in this study, gait measurements could even be performed in an unconstrained setting such as the home environment of patients which would give insights into the actual walking performance of patients during daily life. Furthermore, as the sensor-based gait analysis gives a more comprehensive picture of the walking pattern, potentially shorter measurements could be possible which would further increase the accessibility of these tests for a wider range of patients. Future work could focus on determining the minimum number of steps needed for a robust characterization of the walking pattern using IMUs.

## Conclusions

This work presented a method to identify non-redundant and interpretable gait parameters to characterize walking after a SCI. Gait parameters were derived from a sparse inertial sensor setup, which opens up the possibility of being used within the clinical routine as a technology-aided gait assessment. The extracted gait metrics complemented the standard clinical assessment by providing information related to fatigue, compensatory mechanisms, and rhythm issues. Hence, the diverse gait deficits of this heterogeneous patient cohort could be described more objectively and comprehensively than in the current clinical practice. Further, sensor-derived gait parameters enhanced the prediction of whether a patient will improve his or her walking capacity in the future and exhibited predictors related to improvement in walking capacity. In conclusion, this work is a step towards using sensor-based gait analysis for rehabilitation assessment of patients with a SCI. Such sensor measures could not only foster a more deficit-oriented therapy by providing objective measures on gait deficits but also enhance more targeted rehabilitation plans under consideration of better recovery profile prediction models when including sensor-derived parameters.

## Data Availability

The data presented in this manuscript are available upon reasonable request and under consideration of the ethical regulations.

## Abbreviations

6MWT: Six-minute walking test
ACC: Angular component of coefficient of correspondence
AIS: American Spinal Injury Association Impairment Scale
asym: Asymmetry
BMI: Body mass index
cov: Coefficient of variation
d2r: Difference to reference
HC: Healthy control
IMU: Inertial measurement unit
LEMS: Lower extremity motor score
NLI: Neurological level of injury
PC: Principal components
SCI: Spinal cord injury
SCIM: Spinal cord independence measure
SEM: Standard error of measurement
SSD: Sum of squared differences
WISCI II: Walking index for spinal cord injury
